# StepTest4all: Improving the Prediction of Cardiovascular Capacity Assessment in Young Adults

**DOI:** 10.3390/jfmk9010030

**Published:** 2024-02-08

**Authors:** Tatiana Sampaio, Jorge E. Morais, José A. Bragada

**Affiliations:** 1 Department of Sports Sciences, Instituto Politécnico de Bragança, 5301-856 Bragança, Portugal; tatiana_sampaio30@hotmail.com (T.S.); jbragada@ipb.pt (J.A.B.); 2 Research Center in Sports, Health and Human Development (CIDESD), 6201-001 Covilhã, Portugal; 3 Research Centre for Active Living and Wellbeing (LiveWell), Instituto Politécnico de Bragança, 5301-856 Bragança, Portugal

**Keywords:** StepTest4all, cardiovascular capacity, validation, cardiovascular classification, health

## Abstract

Cardiovascular capacity, expressed as maximal oxygen uptake (VO_2max_), is a strong predictor of health and fitness and is considered a key measure of physiological function in the healthy adult population. The aim of this study was to investigate the influence of the physical activity levels (PA_level_) of participants in the StepTest4all (validated protocol for the estimation of VO_2max_ in adults). The sample consisted of 69 participants, including 27 women (age 21.7 ± 3.6 years; body mass = 63.5 ± 14.8 kg; height = 1.64 ± 0.06 m; body mass index = 23.7 ± 5.3 kg/m^2^) and 42 men (aged 21.7 ± 3.4 years; body mass = 72.0 ± 7.3 kg; height = 1.77 ± 0.07 m; body mass index = 23.1 ± 2.1 kg/m^2^). The participants were assigned to one of the two groups: (i) the VO_2max_ prediction group and (ii) the prediction model validation group. In the multiple linear regression, the following predictors of VO_2max_ remained significant: sex (*p* < 0.001), physical activity level (*p* = 0.014), and HRR_60_ (*p* = 0.020). The prediction equation (R^2^ = 74.0%, SEE = 4.78) showed a close and strong relationship between the measurements and can be expressed as follows: VO_2max_ = 17.105 + 0.260·(HRR_60_) + 8.563·(sex) + 4.097·(PA_level_), in which HRR_60_ is the magnitude of the HR decrease (bpm) in one minute immediately after stopping the step, and sex: men = 1, women = 0, and PA_level_ is level 1 (low), level 2 (moderate), and level 3 (high). The StepTest4all was shown to be a suitable method for estimating cardiovascular capacity, expressed as VO_2max_, in young adults. Retaining PA_level_ as a significant predictor allows us to better individualize the participants’ VO_2max_.

## 1. Introduction

Cardiorespiratory capacity is the ability of the circulatory, respiratory, and muscular systems to deliver and use oxygen during prolonged physical exercise [1]. Expressed as maximal oxygen uptake (VO_2max_), it has emerged over the years as a strong predictor of overall fitness and a key physiological metric in the healthy adult population [2,3,4,5]. Given the abundance of data supporting the importance of cardiorespiratory capacity, the American Heart Association (AHA) issued a scientific statement in 2016 recommending that cardiorespiratory capacity be considered a clinical vital sign [6]. As such, maintaining and assessing cardiorespiratory fitness plays a critical role in preventing health decline in the general population [7].

Exercise testing, a valuable tool for estimating cardiovascular fitness, diagnosing cardiovascular disease, and predicting mortality, involves the assessment of an individual’s physiological response to physical exertion [8,9]. This is especially true when focusing on functional capacity and heart rate dynamics, such as heart rate recovery (HRR) [10,11]. Heart rate recovery is defined as the reduction in heart rate from peak exercise during a stress test to the rate one minute after stopping exercise [12]. This can also be determined at various time points beyond one minute (HRR_60_). These additional time points, such as heart rate recovery at 2 min and 5 min, can provide valuable insight into the longer recovery process [13]. The heart rate (HR) response to increased exercise intensity involves complex regulatory mechanisms. This increase in HR is tightly regulated by the action of central and peripheral mechanisms that project afferent inputs to medullary centers in the brain. These afferent inputs result in an appropriate efferent response of the autonomic nervous system branches, i.e., a decrease in parasympathetic and an increase in sympathetic activity [12]. A delayed decline in HR after exercise is a strong predictor of overall mortality [2,12]. Maximal heart rate (HR_max_) is another critical parameter commonly used in exercise physiology. It represents the highest heart rate that an individual can achieve during intense physical effort and is a key component in several prescription exercise models [14].

Scientific evidence supports the association between HRR and cardiovascular disease prognosis, highlighting the importance of routine HRR recording in clinical practice [15,16]. Among the various testing modalities available, step testing is of particular importance because of its accessibility to the general population and its ability to assess cardiovascular capacity and HR dynamics during exertion and recovery [17,18,19]. However, existing step tests often have limitations, such as pre-determined durations, efforts that exceed recommended levels for certain demographics, and fixed step heights that are inappropriate for certain individuals [20]. For example, the Harvard Step Test [21] and the YMCA Step Test [22] are commonly used, but their fixed protocols may not account for the varying physical abilities and characteristics of individuals. In addition, the Queen’s College Step Test [23] is limited by a fixed duration, which can be challenging for participants with varying fitness levels.

To address these concerns, Bragada et al. [24] introduced the StepTest4all, a cardiovascular capacity assessment protocol that involves participants in a continuous progressive test on a stable step ranging from 15 to 40 cm, alternating between ascending and descending, with a maximum duration of 10 min. The StepTest4all is distinguished for being adaptable, suitable for people with different physical abilities, with a personalized step height and adjustable difficulty to help participants easily reach the target effort level (80% of HR_max_) in a short period of 4 to 10 min. This study retained a significant relationship between VO_2max_, sex and HRR_60_ [24]. However, the authors noted that the lack of a variable related to the amount and intensity of the physical activity of participants could be a limitation [24]. A study conducted by Dyrstad et al. [25] emphasized the effect of physical activity level (PA_level_) on cardiorespiratory capacity. This was accomplished by examining the associations between cardiorespiratory fitness and PA_level_, as measured by both the IPAQ (International Physical Activity Questionnaire) and accelerometers, in a large national sample. Key findings showed that individuals, regardless of sex, who met the physical activity recommendations had 5–13% higher VO_2max_ compared to those who did not meet the recommendations. Similarly, a study by Sharma et al. [26] found a significant increase in VO_2max_ after a program of structured and unstructured physical activity in both female and male participants. Therefore, it is important to recognize the role of PA_level_ as it plays a pivotal role in influencing cardiorespiratory capacity.

In this context, the aim of this study was to build on the foundation laid by Bragada et al. [24] by refining and validating the StepTest4all protocol. By addressing the limitations identified in the previous research, this study seeks to improve the utility of the protocol in assessing cardiovascular capacity in young adults, possibly through a larger sample size and careful inclusion of physical activity variables in the analysis. Therefore, the aim of this study was to investigate the influence of the PA_level_ of participants in the StepTest4all (validated protocol for the estimation of VO_2max_ in adults). Based on the previous assumptions addressed about the importance of PA_level_, it was hypothesized that the PA_level_ would be added as a significant predictor of the VO_2_ concurrently with sex and HRR_60_.

## 2. Materials and Methods

### 2.1. Participants 

The sample consisted of 69 participants, including 27 women (age 21.7 ± 3.6 years; body mass = 63.5 ± 14.8 kg; height = 1.64 ± 0.06 m; body mass index = 23.7 ± 5.3 kg/m^2^) and 42 men (age 21.7 ± 3.4 years; body mass = 72.0 ± 7.3 kg; height = 1.77 ± 0.07 m; body mass index = 23.1 ± 2.1 kg/m^2^). Those with physical limitations that prevented them from ascending or descending the step or those with medical conditions that prevented them from performing moderate physical exertion were not included in the sample recruitment. All participants signed an informed consent form. All protocols were in accordance with the Declaration of Helsinki regarding human research, and the research design was approved by the Polytechnic Ethics Board.

The groups were randomized and consisted of the following: (i) the VO_2max_ prediction equation group and (ii) the prediction model validation group. The characteristics of the participants are shown in Table 1.

### 2.2. Physical Activity Level (PA_level_)

The International Physical Activity Questionnaire, in its short form, was used to assess physical activity. This was carried out in accordance with the official IPAQ classification procedure [27], which divides people into three levels of physical activity and is consistent with the categorization shown in several studies [28,29,30]. These values are determined by multiplying the total PA completed during the week by a weighted approximation and then multiplying the result by the duration (minutes), frequency (per week), and MET intensity (MET-min/week) [30].

The official IPAQ scoring system classifies individuals into three PA_levels_:

- Low Level: Participants whose energy expenditure does not reach PA_level_.
- Moderate Level: (a) Three or more days of vigorous physical activity for at least 20 min per day; or (b) five or more days of moderate, vigorous, or walking for at least 30 min per day; or (c) five or more days of PA per week (moderate, vigorous, walking, or the sum of PA) for at least 600 MET min each week.
- High Level: At least (a) three days of vigorous physical activity with an energy expenditure of 1500 MET-min/week, or (b) complete at least seven days of physical activity that includes walking, moderate PA, and vigorous PA with an energy expenditure of at least 3000 MET-min/week.

This questionnaire was chosen because of its short form. Extended versions have been shown to overestimate the PA_level_ [30]. In addition, it has been developed and tested specifically to determine PA_levels_ in the adult population, particularly in the 15–69 age group [31].

Table 2 shows the PA_levels_ of the participants categorized based on the IPAQ scores. The scores were translated into Low (Level 1), Moderate (Level 2), and High (Level 3).

### 2.3. Data Collection

An electronic scale (Seca 884, Hamburg, Germany) and a digital stadiometer (Seca 242, Hamburg, Germany) were used to measure anthropometric characteristics. A stationary breath-by-breath electronic metabolic device (Cortex, Model MetaLyzer 3B, Leipzig, Germany) was used to monitor HR and VO_2_. A heart rate transmitter (Polar Electro Oy, Kempele, Finland) is part of the device. The device was calibrated with standard gases prior to each test. The standard error for oxygen and carbon dioxide sensors is 0.1%, according to the manufacturer’s handbook.

Each participant’s VO_2_ and HR were continuously monitored as they performed the activities in the following order: rest, StepTest4all protocol, and recovery. The HR and VO_2_ values obtained were used for further analysis: resting values (average of the last minute of rest), values obtained during StepTest4all (average of the last 5 s of each intensity level), and recovery phase (average of the last 5 s of the first minute of recovery). Resting heart rate (HR) and resting VO_2_ were continuously measured while sitting in a quiet, dimly lit room for ten minutes. The participants were not allowed to nap. The last minute values were used for data analysis. In the recovery phase, although HR values were recorded after the first two minutes, only the value from the first minute was considered. The one-minute recovery period was chosen because it has a higher reproducibility [13].

### 2.4. StepTest4all Protocol

**Figure 1:**
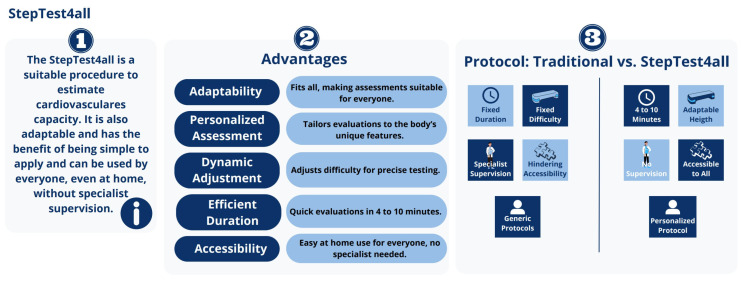
StepTest4all infogram.

Figure 1 shows an infogram of the StepTest4all specificities. Each participant completed a continuous progressive test that involved stepping up and down on a steady step. After the step-up phase, the participant stood vertically, supported by both legs, and the opposite leg also stepped up to the platform. This was followed by the step-down phase. The step-down phase ended when the participant returned to the starting point, where he or she stood vertically again, supported by both legs. It began with the same leg as the previous phase.

Using data on each participant’s cardiovascular capacity, step height was determined for each participant. The variables selected were sex, age, physical fitness, height, body mass index (BMI), and smoking status. Each variable was assigned a numerical value as follows: (i) sex (women = 0.5; men = 1), (ii) age (senior = 0, adult = 0.5, young = 1), (iii) physical fitness (insufficiently active = 0, moderately active = 0.5, vigorously active = 1), (iv) body mass index (BMI < 25 = 0.5, BMI < 30 = 0, BMI ≥ 30 = −0.5), and (v) smoking status (smoker = 0, nonsmoker = 0.5). From these data, the step height was calculated using the formula: step height (cm) = 4 × sum of these variables + 15, and it could range from 15 to 40 cm. These ponderation factors were only used to calculate the step’s height.

This formula is the result of many tests conducted on individuals with varying physical abilities and characteristics. Although the step height is important, it can vary somewhat because the adjustment of the ascent and descent speed is mostly used to control the intensity of the load progression until the appropriate value (80% of the HR_max_) is reached.

In the current study, a step height of 40 cm and a fast pace resulted in an intensity that reached 80% of the estimated maximum heart rate (HR_max_) in 5–10 min. This occurs even in subjects with good physical fitness and tall stature. A height of 40 to 45 cm has been used previously in other protocols, such as the Harvard step [32].

**Figure 2:**
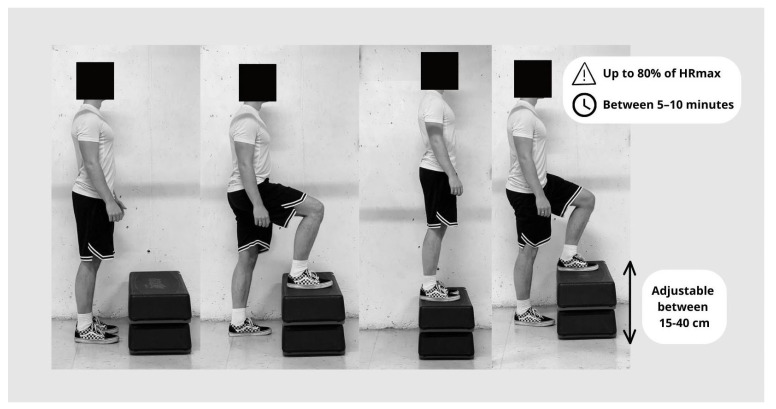
Visual representation of StepTest4all.

Depending on the metronome control, the test began at a rate of 15 cycles per minute (0.25 Hz). In each cycle, the participant walked up and down the step so that the cycle ended at the same time the second leg reached the ground. The cadence was increased by 2.5 cycles per minute. The test should take no more than ten minutes. Anyone can perform the very slow ascent and descent at the lower limit of 15 cycles per minute, which also serves as a warm-up. The maximum speed of 37.5 cycles per minute is limited to subjects with high physical capacity. A visual representation of the StepTest4all is shown in Figure 2.

The test ended when one of the following criteria was met: (i) when the HR reached 80% of HR_max_, (ii) when the subject felt uncomfortable with the exertion, or (iii) when the subject was unable to complete the exercise at the correct cadence. In this case, all participants met the first criterion, i.e., they reached 80% of HR_max_. The participants were instructed to stand for two minutes after completion of the test. While standing, participants were asked not to talk, grab, or hold onto anything. Instead, they were encouraged to relax in order to recover as much as possible.

The step height and rhythm increments, together with the intensity threshold of the test (80% of HR_max_), allowed for the effective assessment of cardiorespiratory capacity in a manageable length of time (5 to 10 min) on a wide range of subjects. HR was continuously collected during the recovery period using the Garmin Fenix 6 and its HR belt (Garmin International, Inc., Olathe, KS, USA).

HR_max_ and VO_2max_ were estimated as follows: HR_max_ was estimated using the formula: HR_max_ = 208 − 0.7·age [33].

Specifically, by determining the value of VO_2_ corresponding to HR_max_, VO_2max_ was estimated using the individual equation of the regression line corresponding to HR–VO_2_ obtained from the resting data and during three or more steps of StepTest4all [34]. This value was assumed when measuring VO_2max_. The range of individual linear regressions (R^2^) was 0.97 to 0.99, indicating an almost perfect relationship. This is a standard and appropriate method for assessing VO_2max_ in those who may find it inconvenient to perform a maximal test to exhaustion.

Submaximal testing has been shown to be an adequate method for estimating VO_2max_ from the HR–VO_2_ relationship [35,36]. In a systematic review, Evans and colleagues [37] reported non-significant discrepancies between the measured and predicted VO_2max_ in 28 equations. HR (N = 19) was the most commonly used variable in the predictive equations. A submaximal treadmill-based protocol was also reviewed by Bennett and colleagues [38]. The authors found that estimating VO_2max_ from the projection of HR_max_ provided a more accurate result.

### 2.5. Statistical Analysis

First, normality and homoscedasticity were assessed using the Kolmogorov–Smirnov and Levene tests, respectively. The means of the descriptive data were computed together with one standard deviation (1 SD). Stepwise regression (backward elimination) was used to predict VO_2max_ based on the following independent variables, i.e., sex, body mass, height, BMI, PA_level_, and HRR_60_. Only significant predictors were retained (*p* < 0.05) in the final model.

The validation procedure between measured and predicted VO_2max_ was based on the following: (i) a comparison of the mean data, (ii) intraclass correlation coefficient (ICC), and (iii) Bland–Altman analysis. The paired samples *t*-test (*p* < 0.05) was used to compare the mean data between the estimated and measured VO_2max_. The effect size index used was Cohen’s d, along with the mean difference and 95% confidence intervals. Cohen’s d was considered to be (i) trivial (<0.20), (ii) small (0.20–0.59), (iii) moderate (0.6–1.19), (iv) large (1.2–1.99), and (v) very large (≥2.0) [39]. The two-way mixed model with an “absolute agreement” definition was used for the ICC [40]. The qualitative interpretation was performed as follows: (i) poor, if ICC < 0.5; (ii) moderate, if 0.5 ≤ ICC < 0.75; (iii) good, if 0.75 ≤ ICC < 0.90; and (iv), excellent, if ICC > 0.90 [40]. Bland–Altman plots showing the mean and difference between the measured and predicted VO_2max_ were analyzed [41]. At least 80% of the plots were considered to be within the ±1.96 standard deviation of the difference (95%CI) for qualitative assessment.

## 3. Results

In the multiple linear regression, the following predictors of VO_2max_ remained significant: sex (*p* < 0.001), PA_level_ (*p* = 0.014) and HRR_60_ (*p* = 0.020). Age, body mass, height, and BMI were not significant in this model. The prediction equation (R^2^ = 74.0%, SEE = 4.78) showed a close relationship between the measurements and can be expressed as follows:

VO_2max_ = 17.105 + 0.260·(HRR_60_) + 8.563·(sex) + 4.097·(PA_level_) (1)

In which VO_2max_ is the maximum oxygen uptake (mL·kg^−1^·min^−1^), HRR_60_ is the heart rate recovery (beats per minute) for one minute immediately after the end of the step test, sex is zero for women and 1 for men, and PA_level_ is level 1 (low), level 2 (moderate), and level 3 (high).

Table 3 shows the comparison between measured and estimated VO_2max_. The results showed nonsignificant differences with a trivial effect size.

**Figure 3:**
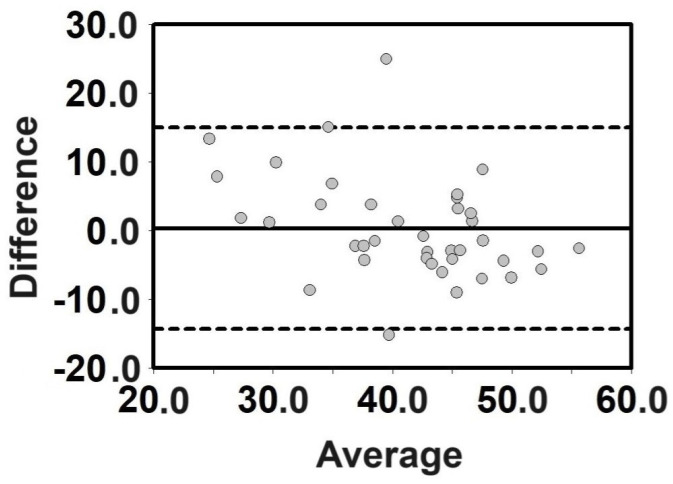
Bland–Altman plots of the measured and predicted VO_2max_. The y-axes refer to the difference between the measured and predicted VO_2max_ [mL·kg^−1^·min^−1^]. The x-axes refer to the average between the measured and predicted VO_2max_ [mL·kg^−1^·min^−1^].

The ICC between measured and predicted VO_2max_ showed good agreement between the measurements (ICC = 0.759, *p* < 0.001). Figure 3 shows the Bland–Altman plots. This analysis also met the agreement criteria with more than 80% of the plots within the 95% CI.

## 4. Discussion

The aim of this study was to investigate the influence of the PA_level_ of participants in the StepTest4all (validated protocol for estimating VO_2max_ in adults). The study retained the PA_level_ as a significant predictor of VO_2max_ simultaneously with the previous predictors (sex, HRR_60_) of the young adult population. In addition, these results show that the magnitude of the heart rate decrease that occurs immediately after exercise is a useful indicator of cardiovascular capacity. This suggests that StepTest4all can be used to assess cardiovascular capacity for individualized, longitudinal monitoring of cardiovascular fitness. Regular use of the StepTest4all facilitates tracking of cardiovascular fitness progression over time. However, comparing VO_2max_ results between different populations should be carried out with caution. The same VO_2max_ value may indicate different physical capabilities for different demographic variables, including age and sex. Therefore, individual VO_2max_ values should be compared with benchmark tables available in the literature to verify compliance with the proposed standards [42].

An attenuated HRR, defined as an insufficient decrease in HR immediately after exercise, indicates decreased parasympathetic nervous system activity [43,44]. The decrease in HR during recovery is mostly caused by the reactivation of the parasympathetic nervous system, which occurs primarily in the initial phase of recovery [45]. Measurement of the post-exercise HR decline also provides an indication of neural system function [46]. Research has shown that a small drop in heart rate in the minutes following the end of exercise is associated with a higher risk of cardiovascular problems [47] and may even be the cause of early mortality [12]. Conversely, a faster decline in HR after exercise is correlated with improved cardiovascular capacity [13]. A study also found that sedentary healthy individuals can improve heart rate recovery (HRR_60_ and HRR_120_) by engaging in moderate-intensity exercise [48].

Adabag and Pierpont’s [49] findings on the recovery of heart rate during exercise are consistent with the current study and emphasize that in recent years, assessments have been used more frequently to evaluate risk and functional autonomic state in both healthy individuals and those with a variety of disorders. HR is usually calculated as the difference (HRR_60_ and HRR_120_, respectively) between the maximum heart rate and the heart rate one to two minutes after stopping exercise. Other measures, including HRR_180_, HRR_240_, and HRR_300_, have also been provided. Short-term reproducibility is demonstrated by these results, and validation has been established for HRR_60_ and HRR_120_. For example, HRR values of 12–13 bpm in 1 min are referred to as threshold levels in a review by Adabag et al. [49]. However, due to the wide variety of tests used and the level of demand, care must be taken when setting cut-off values (between normal and abnormal). It is known that healthy athletes can recover 60 bpm or more in one minute, which is the ideal recovery number. Therefore, values between 12 and 60 bpm can be used to measure the quality of recovery. Increasingly higher values indicate very good cardiovascular capacity and good autonomic nervous system function; values close to 12 bpm may indicate a higher risk of cardiovascular disease or parasympathetic nervous system dysfunction [49,50]. The average HRR_60_ value found in the participants of the present study was 37 ± 11 bpm. This value is well above the minimum values mentioned earlier. In addition, a study by Carnethon et al. [51] found that participants who self-reported a high level of physical activity had a significantly higher HRR (but in this case measured after 2 min of exercise cessation) than participants in the lowest group (corresponding to the lowest level of physical activity). Thus, physical activity was associated with a faster HRR in a treadmill exercise test. Therefore, the participants in the current study seem to be consistent with their age group and active lifestyle [51].

The range of HRR variation (19 to 63 bpm of recovery over one minute) commonly found in these individuals was divided, and four categories were developed to provide a qualitative description of cardiovascular capacity in this population group (young adults). The cardiovascular capacity categorization, the reference VO_2max_ values for the participants in the present study aged between 18 and 29 years, and the values of comparable categories proposed by McArdle et al. [52] are shown in Table 4. This finding may indicate that there is no difference between the VO_2max_ values calculated by Equation (1) and other estimates. Using the StepTest4all, it has been observed that values below 25 are typically associated with a sedentary lifestyle and the presence of additional risk factors such as obesity and smoking, while values above 55 are typically found in individuals who lead a healthy lifestyle and engage in high levels of daily physical activity. Table 4 shows the VO_2max_ values predicted by Equation (1) (from our study) and the values proposed by McArdle et al. [52] for similar categories. Similar values can also be found in a company of world-renowned body composition assessment company (Tanita: https://tanita.eu/blog/could-improving-your-vo2-max-be-the-secret-of-success (accessed on 28 July 2022)) [53].

The assessment of physical activity as a predictor of VO_2max_ is consistent with previous research, such as the study conducted by Dyrstad et al. [25]. The aim of this study was to investigate how different levels of self-reported and objectively measured physical activity, including sedentary time, correlated with variations in VO_2max_. This study included a sample of 759 participants (366 women and 393 men) with a mean age of 48.5 years (SD of 14.4) who completed the cardiopulmonary exercise test 5–8 months after completing the IPAQ questionnaire. The article by Dyrstad et al. [25] examines the relationship between physical activity and cardiorespiratory fitness, both of which are inversely associated with disease and all-cause mortality. Their results indicate that individuals classified as highly active by the IPAQ had higher cardiorespiratory fitness than those who reported low levels of physical activity. In addition, meeting the physical activity recommendation of 150 min per week of daily moderate-intensity physical activity was associated with higher cardiorespiratory fitness. The study highlights the variation in cardiorespiratory fitness and underscores the central role of physical activity in maintaining good health and reducing the risk of disease and mortality. Indeed, our results showed that the PA_level_ was retained as a significant predictor of VO_2max_. In comparison to the study of Bragada et al. [24], our modeling allowed us to increase the prediction output (R^2^ from 63% to 74%). This reinforces the importance of PA_level_ in the assessment of cardiorespiratory capacity.

Because of its unique characteristics, the StepTest4all demonstrates adaptability to individuals with different physical abilities and varying somatic characteristics. While not the first step test to incorporate multiple characteristics in determining step height, this protocol refines this approach. This refined calculation allows for a primary adjustment that prevents the test from becoming too challenging or too easy. Further precision is achieved through careful control of the pace and its incremental adjustments throughout the test to ensure that the desired intensity of effort associated with 80% of HR_max_ (upper limit) is achieved within an appropriate time frame of 5 to 10 min for all participants. This updated protocol builds on the foundation laid by Bragada et al. [24], contributing advancements to the methodology and enhancing its effectiveness in assessing cardiovascular capacity among individuals with varying physical characteristics.

In the current study, age was excluded as a predictor of VO_2max_ via stepwise regression modeling. This exclusion was influenced by the homogeneity of the sample, which consisted predominantly of young adults. It is plausible that in studies with a more diverse age range, age could become an important factor in the predictive equation for VO_2max_. Expanding the age spectrum in future investigations may shed light on the potential impact of age on the predictive accuracy of the model.

In addition to this, the use of step tests, coupled with the above-mentioned advantages, remains favorable due to their simplicity, minimal space requirements, and ability to be performed by individuals at home. As demonstrated on the StepTest4all Facebook page (https://www.facebook.com/StepTest4all, accessed on 28 July 2022) [54], the accessibility of the protocol enhances its practicality and convenience, making it an attractive option for widespread participation in cardiovascular fitness assessments.

The current study has several limitations that need to be considered. First, the research was conducted with young adults only. Therefore, further studies with a broader demographic representation are needed to generalize the findings to different age groups. Second, it is important to note that both VO_2_ and HR_max_ values were estimated rather than directly measured. However, these estimates were derived from the evolution of actual individual values of VO_2max_ and HR, both at rest and at different intensity levels. It is important to emphasize that this estimation method is consistent with standard procedures commonly used in non-athletic participants or special populations where it is not advisable to subject individuals to maximal tests to exhaustion. Finally, the reliability of the test was not measured in this particular sample. Thus, further studies could address this issue. Despite these limitations, the findings of this study provide a foundation for future research efforts aimed at addressing these limitations and expanding the applicability of the StepTest4all protocol.

## 5. Conclusions

The StepTest4all was shown to be a suitable method for estimating cardiovascular capacity, expressed as VO_2max_, in young adults. The validation procedure showed a high degree of agreement between measured and estimated VO_2_. Additionally, it is possible to determine the qualitative level of cardiovascular capacity (PA_level_) from the HRR_60_, more specifically, poor: <25 bpm, moderate: 25 to 39 bpm, good: 40 to 55 bpm, and excellent: ≥55 bpm. This method is easy to use and accessible to everyone, so it can be used at home without the need for specialized supervision.

## Figures and Tables

**Table 1:** Characteristics of the participants.

	Equation Group			Validation Group		
	Mean ± 1 SD			Mean ± 1 SD		
	Women (n = 17)	Men (n = 13)	Total	Women (n = 10)	Men (n = 29)	Total
Age [years]	20.8 ± 2.3	21.6 ± 2.8	21.7 ± 2.4	23.1 ± 4.8	21.7 ± 3.6	22.1 ± 3.9
Body mass [kg]	57.0 ± 6.8	71.6 ± 7.0	68.7 ± 11.5	74.6 ± 18.2	72.2 ± 7.6	71.8 ± 11.0
Height [m]	1.61 ± 0.04	1.77 ± 0.07	1.72 ± 0.09	1.68 ± 0.07	1.77 ± 0.07	1.74 ± 0.08
BMI [kg/m^2^]	22.0 ± 3.1	22.8 ± 1.6	23.3 ± 3.7	26.7 ± 6.9	23.2 ± 2.3	24.1 ± 4.2
HRR_60_ [bpm]	36 ± 9	36 ± 11	36 ± 10	37 ± 10	38 ± 11	38 ± 11
VO_2max_ [mL·kg^−1^·min^−1^]	32.86 ± 4.95	45.03 ± 8.19	39.83 ± 9.38	32.95 ± 9.20	43.97 ± 8.19	41.14 ± 9.65
HRR_rest_ [bpm]	83 ± 11	67 ± 11	76 ± 13	78 ± 12	69 ± 11	71 ± 12
VO_2rest_ [mL·kg^−1^·min^−1^]	3.53 ± 0.56	3.97 ± 0.67	3.73 ± 0.64	3.17 ± 0.92	3.44 ± 0.61	3.37 ± 0.70

Note: Total—both sexes summed together.

**Table 2:** Physical activity levels (PA_levels_) of the participants.

	Equation Group		Validation Group	
	Women (n = 17)	Men (n = 13)	Women (n = 10)	Men (n = 29)
PA_level_				
1	8	0	7	3
2	6	7	2	17
3	3	6	1	9

Note: PA_level_—physical activity level.

**Table 3:** Paired samples *t*-test comparison between measured and estimated VO_2max_ in the validation group. The effect size index (Cohen’s d) is also shown.

Measured VO_2max_ [mL·kg^−1^·min^−1^]	Estimated VO_2max_ [mL·kg^−1^·min^−1^]			
Mean ± 1 SD	Mean ± 1 SD	Mean Difference (95% CI)	*t*-Test (*p* Value)	d [Descriptor]
41.14 ± 9.65	41.48 ± 6.94	−0.345 (−2.767 to 2.076)	−0.289 (0.774)	0.04 [trivial]

Note: VO_2max_: maximal oxygen uptake.

**Table 4:** Cardiovascular capacity (CVC) classification based on HRR_60_ and corresponding HRR cut-off values.

CVC Classification	HRR_60_	Men		Women	
		VO_2max_	McArdle et al., 2003 [52]	VO_2max_	McArdle et al., 2003 [52]
Poor	<25	<40	<36.5	<28	<29
Moderate	25–39	42–44.2	36.5–42.4	28–32.2	29–32
Good	40–54	44.3–49	42.5–46.4	32.3–36.9	33–36
Excellent	≥55	≥49	≥46.5	≥37	≥37

## Data Availability

Data are available upon request to the contact author.
